# Supplementary material for: The Systems Biology Research Tool: evolvable open-source software
Source: BMC Syst Biol. 2008 Jun 29;2:55. doi: 10.1186/1752-0509-2-55 (PMC2446383; doi:10.1186/1752-0509-2-55)
Supplement: Additional file 1 — SBRT Archive. An archive of the current version of the Systems Biology Research Tool. [file 1752-0509-2-55-S1.zip › sbrt-1.4.0/doc/developers_guide/api/sbrt/kernel/fba/proc/class-use/FbaProcess.html]

Uses of Class sbrt.kernel.fba.proc.FbaProcess


|  |  |  |
| --- | --- | --- |
| **Overview** \| **Package** \| **Class** \| **Use** \| **Tree** \| **Deprecated** \| **Index** \| **Help** | |  |
| PREV   NEXT | **FRAMES**    **NO FRAMES**     **All Classes** |  |


---


## **Uses of Class sbrt.kernel.fba.proc.FbaProcess**

| Packages that use FbaProcess | |
| --- | --- |
| **sbrt.kernel.fba.proc** | Provides classes and interfaces for executing Flux Balance Analysis processes. |
| **sbrt.shell.mng.fba** | Provides classes and interfaces for creating Flux Balance Analysis process managers. |

Uses of FbaProcess in sbrt.kernel.fba.proc

| Subclasses of FbaProcess in sbrt.kernel.fba.proc | |
| --- | --- |
| `class` | `CatalystDeletionProcess`             This class is used to perform catalyst deletion experiments. |
| `class` | `CdHarFluxVectorProcess`             This class is used to perform coordinate direction hit-and-run flux vector experiments. |
| `class` | `CvCatalystDeletionProcess`             This class is used to perform constraint-variation catalyst deletion experiments. |
| `class` | `CvFbaOptimizationExperiment`             This class is used to perform constraint-variation FBA optimization experiments. |
| `class` | `CvFbaOptProcess<R>`             This class is used to represent constraint-variation Flux Balance Analysis experiments. |
| `class` | `CvFluxIntervalProcess`             This class is used to perform constraint variation flux interval experiments. |
| `class` | `CvObjectiveFunctionProcess`             This class is used to perform constraint-variation objective function experiments. |
| `class` | `CvRxnDeletionProcess`             This class is used to perform constraint-variation reaction deletion experiments. |
| `class` | `CycleIdProcess`             This class is used to execute steady state cycle identification processes. |
| `class` | `ExtremeCurrentProcess`             This class is used to perform extreme current experiments. |
| `class` | `FbaOptimizationProcess`             This class is used to perform basic Flux Balance Analysis optimization. |
| `class` | `FbaSolverProcess`             This class is used to solve Sv = 0. |
| `class` | `FluxActivityProcess`             This class is used to perform flux activity experiments. |
| `class` | `FluxCapProcess`             This class is used to perform a flux cap identification. |
| `class` | `FluxIntervalProcess`             This class is used to perform flux interval experiments. |
| `class` | `FluxomeReducerProcess<T extends Fluxome>`             This class is used to execute fluxome reduction processes. |
| `class` | `FluxPlasticityProcess`             This class is used to perform flux plasticity experiments. |
| `class` | `InitialPointGenerator`             This class is used to perform initial point experiments. |
| `class` | `NetworkInfoProcess`             This class is used to gather basic information about a fluxome. |
| `class` | `ObjectiveFunctionAnalysis`             This class is used to perform objective function experiments. |
| `class` | `RandomConstraintsGeneratorV1`             This class is used to generate random flux constraints. |
| `class` | `RandomEnvGeneratorV1`             This class is used to generate random environments for use in flux balance analysis. |
| `class` | `RandomObjectiveGenerator`             This class is used to generate random objective functions. |
| `class` | `RxnDeletionProcess`             This class is used to perform reaction deletion experiments. |
| `class` | `StoichVariationProcess`             This class is used to perform stoichiometry-variation FBA optimizations. |
| `class` | `SvCdHarFluxVectorProcess`             This class is used to perform space variation coordinate direction hit-and-run flux vector experiments. |
| `class` | `SvInitialPointProcess`             This class is used to perform constraint variation initial point experiments. |

Uses of FbaProcess in sbrt.shell.mng.fba

| Methods in sbrt.shell.mng.fba with parameters of type FbaProcess | |
| --- | --- |
| `static ApplicationException` | `FbaOptManager.interpret(ConstraintViolationException e, FbaProcess<?> experiment)`             Returns an `ApplicationException` whose message describes the error that ocurred. |
| `static ApplicationException` | `FbaOptManager.interpret(FbaOptException e, FbaProcess<?> experiment)`             Returns an `ApplicationException` whose message describes the error that ocurred. |
| `static ApplicationException` | `MsFluxIntervalManager.interpret(FluxRangeException e, FbaProcess experiment)`             Returns an `ApplicationException` whose message describes the error that ocurred. |
| `static ApplicationException` | `FbaOptManager.interpret(java.lang.RuntimeException e, FbaProcess<?> experiment)`             Returns an `ApplicationException` whose message describes the error that ocurred. |

---


|  |  |  |
| --- | --- | --- |
| **Overview** \| **Package** \| **Class** \| **Use** \| **Tree** \| **Deprecated** \| **Index** \| **Help** | |  |
| PREV   NEXT | **FRAMES**    **NO FRAMES**     **All Classes** |  |


---
